# Effect of Composition Strategies on Mycelium-Based Composites Flexural Behaviour

**DOI:** 10.3390/biomimetics7020053

**Published:** 2022-04-25

**Authors:** Adrien Rigobello, Claudia Colmo, Phil Ayres

**Affiliations:** Centre for IT and Architecture, Royal Danish Academy, 1435 Copenhagen, Denmark; ccol@kglakademi.dk (C.C.); phil.ayres@kglakademi.dk (P.A.)

**Keywords:** mycelium-based composite, biomaterials, natural composites

## Abstract

Mycelium-based composites (MBC) are a promising class of relatively novel materials that leverage mycelium colonisation of substrates. Being predicated on biological growth, rather than extraction based material sourcing from the geosphere, MBC are garnering attention as potential alternatives for certain fossil-based materials. In addition, their protocols of production point towards more sustainable and circular practices. MBC remains an emerging practice in both production and analysis of materials, particularly with regard to standardisation and repeatability of protocols. Here, we show a series of flexural tests following ASTM D1037, reporting flexural modulus and flexural modulus of rupture. To increase the mechanical proprieties, we contribute with an approach that follows the composition strategy of reinforcement by considering fibre topology and implementing structural components to the substrate. We explore four models that consist of a control group, the integration of inner hessian, hessian jacketing and rattan fibres. Apart from the inner hessian group, the introduction of rattan fibres and hessian jacketing led to significant increases in both strength and stiffness (α = 0.05). The mean of the flexural modulus for the most performative rattan series (1.34 GPa) is still close to three times lower than that of Medium-Density Fibreboard, and approximately 16 times lower in modulus of rupture. A future investigation could focus on developing a hybrid strategy of composition and densification so as to improve aggregate interlocking and resulting strength and stiffness.

## 1. Introduction

Mycelium-based composites (MBC) are a novel field of material development leveraging wood-decaying basidiomycota to bind lignocellulosic particulate media via Solid-State Fermentation (SSF) [1]. Since 2006 and the establishment of the first commercial venture for MBC products (Ecovative Design, LLC, Green Island, NY, USA) the development of these materials has been supporting packaging and insulation use cases. Both these cases are relevant with a state-of-the-art that has favoured local replication of the production process across the globe and across institutions before advancing studies of the material mechanical model for assessing alternative use cases. The variety of versatile fungi that can be used, coupled with the extensive variety of lignocellulosic substrates with respect to aggregate geometries and chemical profiles, supports a wide design space. The spread of the MBC state-of-the-art regarding only reported stiffness and strength is a strong testimony to this, while research has yet to lead to functional poles definition [1]. Furthermore, the principal advantage of MBC designs lies in their higher potential for upcycling agricultural wastes, leading to the production of resource conscious and biodegradable materials with a low environmental impact [2] that can contribute to shifting towards a circular economy.

Across the literature, three principal design strategies for modifying MBC mechanical behaviour have been identified: densification, composition, and supplementation (targeting mycelium properties, based on chemical tuning of the substrate) [1]. Mechanically, MBC have been investigated mainly as per their compressive and flexural behaviour [1]. Densification is a strategy for stiffening the composite by increasing the density of the substrate by dense packing, cold or hot pressing. Densified specimens have been reportedly leading to an increase of flexural modulus between 27 and 72-fold, and 4 to 14.5-fold increase in flexural strength for a densification from 100–130 kg/m^3^ to 350–390 kg/m^3^ [3]. While staying at the lower end of MBC densities, another study reported an increase of 4.4-fold in flexural strength while increasing composite density by a factor 1.4 (from 102 to 141 kg/m^3^) [4]. Composition is a second strategy for modifying MBC behaviour by adding structural components to the substrate, including, for instance, orientated fibres or textiles. Modifying particle properties is also considered an instance of the composition strategy [1]. The MBC state-of-the-art largely considers monolithic and homogeneous composites, besides a few study groups investigating jute type materials in sandwich composite reinforcement, and wood panels introduction [5,6,7]. Composition strategies by arming or particulate design are therefor a scarcely studied area of material development still, while the lower stiffness of the mycelial matrix (the tensile modulus of *Pleurotus ostreatus* and *Ganoderma lucidum* species is reportedly in the 4–28 MPa range [8]) as compared to, for instance, American beech wood (*Fagus grandifolia*) elastic modulus of 11.9 GPa at 12% moisture content (MC) [9], suggests that the design of the composite dispersed phase can considerably influence the final composite mechanical stress response. The significance of composition strategies over the composite compressive behaviour has been reported previously, both for aggregate size and fibre placement [10].

Three studies have been investigating the effect of composition strategy on MBC flexural behaviour. A hybrid protocol using both composition and densification techniques has been employed by using blend bacterial cellulose (BC) produced by a *Komagataeibacter xylinus* colony. The BC cellulose fibrils were mixed to the hemp fibres serving as principal substrate, and set to be colonised by a *Trametes versicolor* species [11]. This method targets an increase in aggregates binding. BC introduction did not result in a statistically significant difference. Nonetheless, pressing temperature change from 70 °C to 200 °C led to a 1.42-fold increase in stiffness and a factor 1.54 increase in flexural strength to reach 2.94 MPa. The two remaining relevant studies have studied the effect of textile lamination on top and bottom surfaces of a composite. One of them reported flexural moduli in 4.65–6.57 MPa, with moduli of rupture in 0.76–1.5 MPa, without reporting on the statistical significance of the different lamination materials used neither on the fungal species that the material was cultivated with [5]. Results of a greater stiffness have been reported with the introduction of top and bottom carbon-fibre layers, leading to a modulus of 296 MPa for a modulus of rupture of 2.9 MPa. This last study also investigated bamboo lamination and saw a stiffness increase of a factor 2.18, while flexural strength dropped to 0.31 times the carbon fibre laminated composite group. No density was reported for the specimens in this study, neither proprietary supplements to the substrate [12].

In the study reported here, we focus on the effect of composition over the flexural behaviour with the introduction of orientated fibres and hessian. Following ASTM D1037, we report on three point bending for three categories of composition: the embedding of a hessian arming at mid-thickness, hessian jacketing, and rattan arming in specimen length. Apart from the inner hessian group (BM_I), the introduction of rattan fibres and hessian jacketing led to significant increases in both strength and stiffness (α = 0.05).

## 2. Materials and Methods

Referenced standards for evaluating flexural properties of MBC in the state-of-the-art are presented in Table 1. ASTM D1037 was used as the standard evaluation method [13]. We use this standard because it is the most referred set of guidelines in MBC development, covers various tests and refers to the fittest material model. We report on three point bending. To this end, four specimen groups were designed:

- Control: no fibre,
- Inner hessian: a flat layer of hessian was introduced at mid-thickness,
- Hessian jacketing: a hessian jacketing was introduced in the length,
- Rattan: five parallel rattan fibres of 5 mm diameter by 500 mm, separated by 8 mm, were introduced in the length and at mid-thickness.

**Figure 1:**
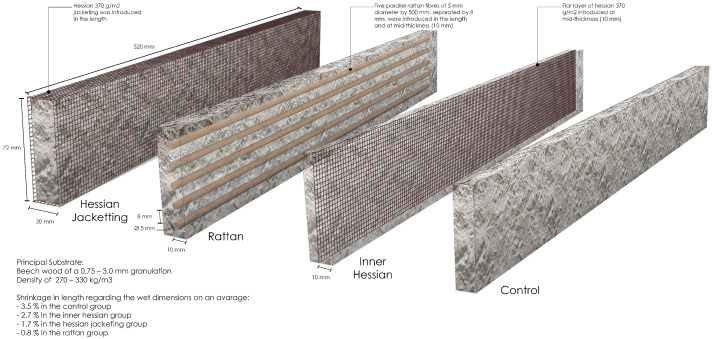
Fibre placement strategies (left to right): hessian jacketing (BM_H), rattan fibres (BM_R), inner hessian (BM_I), control (BM).

The wet specimens are parallelepipeds of 520 mm × 72 mm, with a nominal thickness of 20 mm. The width and thickness were not affected by the desiccation, but the length of the dry specimens varied between reinforcement strategies and shrank on average by 3.5% in the control group, 2.7% in the inner hessian group, 1.7% in the hessian jacketing group, and 0.8% in the rattan group. The distance of the top and bottom specimen surfaces to the neutral axis of stress in flexion was of 10 mm. Six replicates were produced and tested for each of the specimen types. Load testing was performed on a Mecmesin MultiTest-dV testing bench equipped with a 2500 N load sensor, with a loading speed of 10.0 mm/min. Flexural modulus and modulus of rupture were calculated following ASTM D1037. The four specimen groups specifics are illustrated in Figure 1.

### 2.1. Materials

A millet-grown spawn of species *G. lucidum* (reference M9726) was acquired from Mycelia BVBA (Nevele, Belgium). The spawn was stored at a constant 4 °C and 65% RH prior to being used. The principal substrate of the specimens is European beech wood (*Fagus sylvatica*) of a 0.75–3.0 mm granulation (Räuchergold type HB 750/2000, J. Rettenmaier & Söhne GmbH + Co KG, Rosenberg, Germany), and nominal density in 270–330 kg/m^3^. Longitudinal reinforcement was introduced in the BM_R specimens group by using 5 mm diameter rattan fibres (*Calamus manan*; B.V. INAPO, Bloemendaal, Netherland), and hessian was used for the BM_I and BM_H groups (*Cannabis sativa* subsp. *sativa*; NEMO Hemp jam web 370 g/m^2^, Naturellement Chanvre, Echandelys, France).

### 2.2. Cultivation Protocol

The principal substrates, fibres and hessian were prepared at 40% moisture content (MC) with mineralized water and sterilised at 121 °C for 15 min. The principal substrates were then mixed with 16 wt% spawn and incubated in polypropylene filtered bags (SacO2, Deinze, Belgium) for 7 days at 27 °C in the dark. Once colonised, the principal substrates were broken down and formed with the sterile fibre and hessian into alcohol cleaned aerated PETG moulds. The formed specimens were incubated for 21 days at 27 °C in the dark, then oven-dried for 48 h at 60 °C. The dried specimens were stored at 4 °C and 65% RH prior to testing. No external mycelium was cultivated on the boundaries of the specimens. No additives were used.

## 3. Results and Discussion

**Figure 2:**
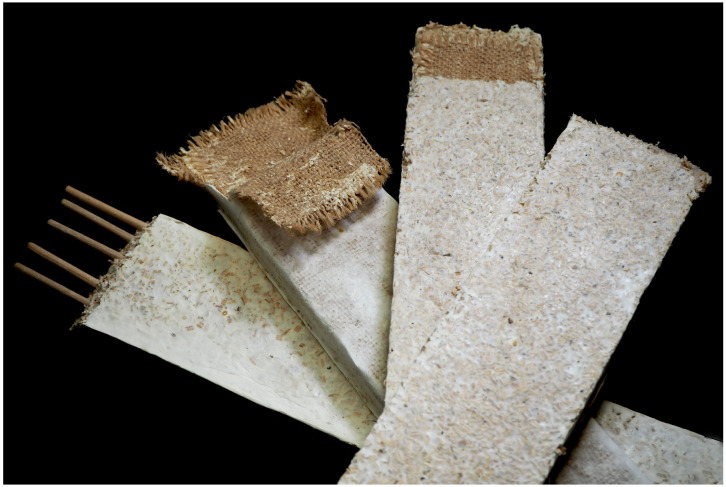
Fibre placement strategies in tested specimens (left to right): rattan fibres (BM_R), hessian jacketing (BM_H), inner hessian (BM_I), control (BM).

**Figure 3:**
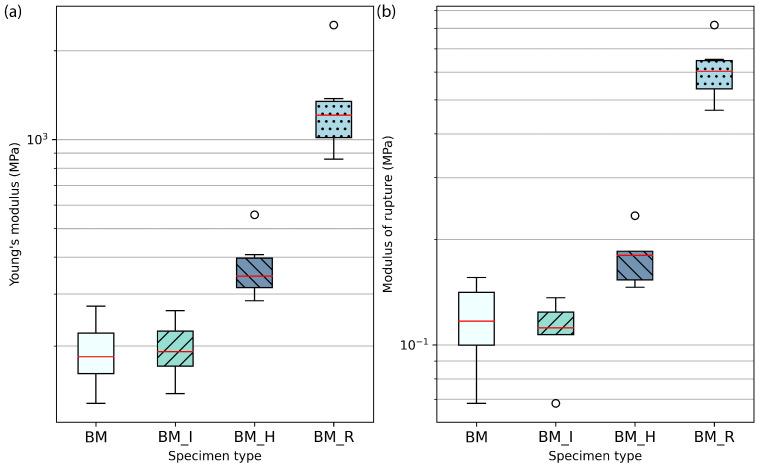
Flexural modulus (**a**) and modulus of rupture (**b**) box plots results.

**Figure 4:**
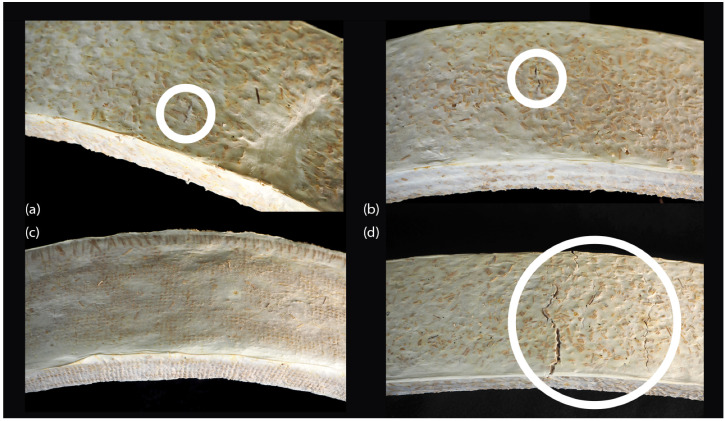
Representative failure modes for the (**a**) BM group, (**b**) BM_I group, (**c**) BM_H group, (**d**) and BM_R group.

We investigated the effect of diverse reinforcements on the mechanical behaviour in flexion of MBC using four levels: control (BM), inner hessian (BM_I), hessian jacketing (BM_H), and rattan fibres in the length (BM_R). In Figure 2 we can observe the dissected specimens after testing. Isotropic controls were added to the experimental series (BM). Experimental parameters per specimen type and resulting mean density, mean flexural modulus and mean modulus of rupture are presented in Table 2. Box plots of the results for flexural modulus and modulus of rupture are presented in Figure 3. It can be noted that the mechanical failure of the specimens was related to dewetting of the principal substrate, beech wood particles, across all groups and at the level of the highest tensile stress, that is at the middle of the span on the opposite surface to the applied load. Rattan fibres did not fail nor deform plastically. Likewise, no debonding of the hessian jacketing was visible after testing. Representative failure modes are presented in Figure 4, where we can appreciate the limited fractures in the most elastic specimen groups BM and BM_I where the mycelial matrix also deformed plastically on the surface that was the most exposed to tensile stress (10 mm from the neutral axis), and the lack of external fracture in the BM_H specimen group. The BM_R group resulted in more external fractures as the aligned continuous rattan fibres were favourable to increasing strength and stiffness while its continuous interfacial bond between the matrix and the rattan fibres constrained the deformation. A future investigation may focus on displacing the fibre reinforcement towards the most stressed opposite surface to the load, distancing it from the neutral axis to improve its efficiency. A backdrop we could expect from this is the earlier formation of fractures on the surface exposed to tensile stress, while the fibre alignment with the neutral axis in this experimental series left a thicker sectional area of beech wood and mycelium complex under the fibres, which allowed for the mycelial matrix to deform elastically and plastically to a greater strain.

Similarly to our previous investigation on compression behaviour characterisation [10], the hessian jacketing series offers a contrasting instance of the effect that the cultivation of an external mycelial skin on test specimen might lead to. The BM_H series resulted in a 1.95-fold increase in flexural modulus as compared to the control group, and 1.5-fold increase in strength. In contrast to the BM_I series which had a hessian reinforcement added at mid-thickness and along the neutral stress axis, jacketing is a particularly suitable fibre composition strategy as the low elasticity of hessian contributes to resisting the tensile stress on the surface opposite to the load and on covered side surfaces. Beech wood particles further contribute to the composite strength where subjected to compression—on the surface where the load is applied—as the elastic mycelial matrix reaches its maximal strain and beech particles of a higher stiffness interlock. The BM_R series has the highest relative standard deviation with 41.3% of the mean, while other groups have a standard deviation of a maximum of 27.2%. An extremum is reported at 2.44 GPa while Q3 is 1.35 GPa for a mean at 1.34 GPa. If not considering this extremum, the mean of the series is at 1.12 GPa for a standard deviation closer to MBC standards at 211.06 MPa (18.8%).

**Figure 5:**
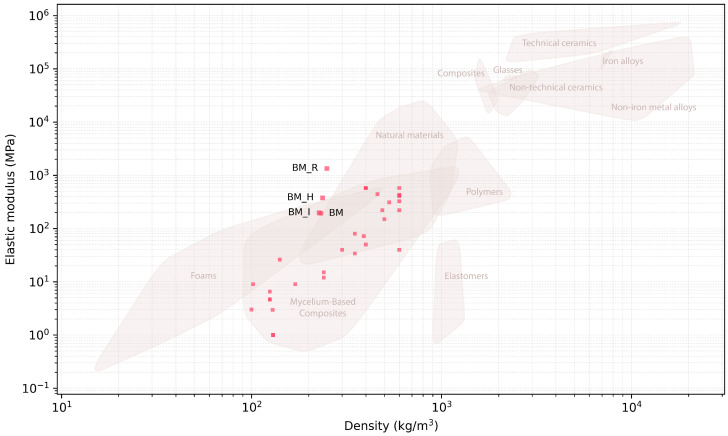
Flexural modulus results as a function of density. Labelled data points: results from this study; unlabelled data points: reports from the state-of-the-art.

**Figure 6:**
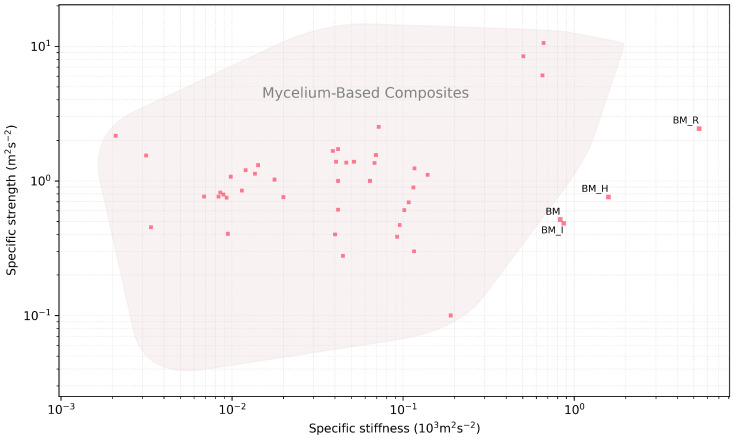
Specific strength results as a function of specific stiffness. Labelled data points: results from this study; unlabelled data points: reports from the state-of-the-art.

The results of the experimental series are plotted on an Ashby map (Figure 5) and are presented as normalised by density (Figure 6). In the latter figure we can notice the increased mechanical efficiency of the BM_R series thanks to the composition strategy. In both figures flexural reports from the published MBC state-of-the-art are plotted [3,4,5,11,12,18,19,20,21,22]. These two figures gather evidences produced with approximately ten fungal species, two studies having not disclosed the ones they used [5,20]. There are 42 data points gathered from ten journal and conference articles. These include articles reporting on strength and/or stiffness in flexion; 3 data points had no density reported [12]. Only the reports with sufficient data are rendered on the figures.

### Statistical Analysis

The result distributions are two-tailed. The mean of Fisher’s defined kurtosis for flexural modulus series is −0.7243 (s.d. 0.6040) and −0.5844 for modulus of rupture (s.d. 0.5019). Fisher-Pearson’s skewness coefficient mean for flexural modulus is 0.5582 (s.d. 0.4907), and −0.1625 for modulus of rupture (s.d. 0.7697). The distributions are considered normal [23], but did not satisfy the Shapiro-Wilk test (modulus: *p* = 1.1266 × 10${}^{-5}$, modulus of rupture: *p* = 3.4568 × 10${}^{-5}$, α = 0.001). Equality of variances was controlled with the Levene test; flexural modulus result variances are equal (*p* = 0.0208, α = 0.05), such as modulus of rupture ones (*p* = 0.0146, α = 0.05). One-way ANOVA was conducted for flexural modulus and modulus of rupture regarding reinforcement strategies (respectively *p* = 1.8619 × 10${}^{-6}$ and *p* = 2.8678 × 10${}^{-11}$). The mean values are significantly different (α = 0.001). Using the pairwise Games-Howell test we confirm the significant difference between the rattan group and the other groups for flexural modulus and modulus of rupture (α = 0.05). The inner hessian group was not significantly different from the control (*p* = 0.900).

## 4. Conclusions

The investigation presented in this paper has focused on the use of natural fibre composition in MBC as a means to modify flexural behaviour. We have demonstrated three fibre composition designs and show significant increases in stiffness and strength comparing the BM_R and BM_H series to the BM control group (α = 0.05). The BM_I group, with a layer of hessian inserted at mid-thickness of the specimens, did not result in a significant effect. The mean of the flexural modulus for the most performative BM_R series (1.34 GPa) is still close to three times lower than that of Medium-Density Fibreboard (MDF; 4 GPa), and approximately 16 times lower in modulus of rupture (0.62 MPa, MDF: 10 MPa). Considering the higher density of MDF (750 kg/m^3^) as compared to the BM_R series (249.48 kg/m^3^) a future investigation could focus on developing a hybrid strategy of composition and densification so as to improve aggregate interlocking and resulting strength and stiffness. Future investigations may also focus on various soft arming positioning strategies reflecting on the most mechanically demanding areas of the specimens as per a defined load case, and use of orientated continuous fibres for a principal substrate. Composite production accuracy improvements are expected to contribute to reducing the standard deviation of results. Furthermore, reinforcements may be strategised in developing efficient or multi-functional composites, for instance, in designing the principal substrate to be thermally performative in addition to introducing reinforcements to perform structurally. We report on specimens dimensional stability after drying linked to the diverse compositions, this aspect can be investigated in the future both as a means to explore design consequences, and for production control.

Considering MBC higher vernacular potential thanks to the versatility and diversity of fungal species that can be used for cultivating, and to meet the urgent sustainability agenda, the sourcing of the raw materials and substrates for MBC should consider local and opportunistic supplies. In this study we make use of Austrian beech wood, hessian manufactured and cultivated in France, and rattan fibres which are produced in South-East Asia and West Afrika countries [24]. Beyond the interest of rattan fibres for their very consistent supply for supporting reproducibility of results and material homogeneity, future market-orientated MBC developments may focus on constraining the geographical footprint of their attached MBC supply chains so to reduce their embedded energy and global resources stress.

## Figures and Tables

**Table 1:** Referenced standards for flexural characterisation in the MBC state-of-the-art.

Standard	Designation	Refs.
ASTM C203	Standard Test Methods for Breaking Load and Flexural Properties of Block-Type Thermal Insulation.	[14]
ASTM C393	Standard Test Method for Core Shear Properties of Sandwich Constructions by Beam Flexure	[5]
ASTM D7250	Standard Practice for Determining Sandwich Beam Flexural and Shear Stiffness	[5]
ASTM D1037	Standard Test Methods for Evaluating Properties of Wood-Base Fiber and Particle Panel Materials.	[15,16,17]
ISO 16978	Wood-based panels—Determination of modulus of elasticity in bending and of bending strength.	[18]
ISO 12344	Thermal insulating products for building applications—Determination of bending behavior.	[18]

**Table 2:** Summary of specimen types parameters, resulting dried densities, and flexural properties.

Type	Fibre Composition	Mean Density (s.d.) [kg/m^3^]	Mean Flexural Modulus (s.d.) [MPa]	Mean Modulus of Rupture (s.d.) [MPa]
BM	Control	232.24 (18.24)	192.71 (52.40)	0.12 (0.03)
BM_I	Inner hessian	227.22 (8.46)	197.33 (45.56)	0.11 (0.02)
BM_H	Hessian jacketing	236.75 (12.00)	375.14 (98.81)	0.18 (0.03)
BM_R	Rattan	249.48 (9.78)	1.34 × 10${}^{3}$ (570.68)	0.61 (0.12)

## Data Availability

The data presented in this study are available on request from the corresponding author.
